# DFMA: an improved DeepLabv3+ based on FasterNet, multi-receptive field, and attention mechanism for high-throughput phenotyping of seedlings

**DOI:** 10.3389/fpls.2024.1457360

**Published:** 2025-01-16

**Authors:** Liangquan Jia, Tao Wang, Xiangge Li, Lu Gao, Qiangguo Yu, Xincheng Zhang, Shanlin Ma

**Affiliations:** ^1^ School of Information Engineering, Huzhou University, Huzhou, China; ^2^ School of Electronic Information Engineering, Huzhou College, Huzhou, China; ^3^ Institute of Crop Science, Huzhou Academy of Agriculture Sciences, Huzhou, China

**Keywords:** plant seedlings, deep learning, plant seedling phenotyping analysis, DeepLabv3+, DFMA

## Abstract

With the rapid advancement of plant phenotyping research, understanding plant genetic information and growth trends has become crucial. Measuring seedling length is a key criterion for assessing seed viability, but traditional ruler-based methods are time-consuming and labor-intensive. To address these limitations, we propose an efficient deep learning approach to enhance plant seedling phenotyping analysis. We improved the DeepLabv3+ model, naming it DFMA, and introduced a novel ASPP structure, PSPA-ASPP. On our self-constructed rice seedling dataset, the model achieved a mean Intersection over Union (mIoU) of 81.72%. On publicly available datasets, including Arabidopsis thaliana, Brachypodium distachyon, and Sinapis alba, detection scores reached 87.69%, 91.07%, and 66.44%, respectively, outperforming existing models. The model generates detailed segmentation masks, capturing structures such as the embryonic shoot, axis, and root, while a seedling length measurement algorithm provides precise parameters for component development. This approach offers a comprehensive, automated solution, improving phenotyping analysis efficiency and addressing the challenges of traditional methods.

## 1 Introduction

“High-Throughput Phenotyping” is a method for rapidly and automatically acquiring and analyzing large volumes of phenotypic data from plant or biological samples. This approach utilizes imaging technology, sensors, computer vision, and machine learning to collect extensive data without disrupting sample growth, thus revealing growth characteristics, health status, and physiological changes of the organisms. This technique is particularly applicable in agriculture and plant sciences, enabling efficient evaluation of different genotypes under various environmental conditions, and providing essential data to support crop improvement and breeding programs. In recent years, plant phenotyping has emerged as a rapidly advancing, data-intensive field (Zhao et al., 2019; Yang et al., 2020). Studying plant phenotypes allows for a deeper understanding of genetic information (Richard et al., 2015; Holman et al., 2016) and the growth trends of plants. When it comes to monitoring the growth of plant seedlings, phenotypic analysis of seedlings becomes particularly crucial. Assessing various aspects of seedling development often requires the measurement of specific physical dimensions, with the length of the hypocotyl being a key phenotypic trait for monitoring and quantifying different responses (Dobos et al., 2019). Hypocotyl cells are formed during embryogenesis and undergo several rounds of cell division to develop. During seedling growth, the length of the hypocotyl is no longer determined by cell division but rather by the elongation of hypocotyl cells (Gendreau et al., 1997). Phenotypic analysis of the root system, known as Root System Architecture (RSA), is also a vital indicator for assessing seedling development. RSA refers to the spatial arrangement of the root system and its components (Lynch, 1995), and its functions include water and nutrient absorption, storage, as well as anchoring and facilitation of plant-microbe interactions, such as nodule formation in nitrogen-fixing crops. Although these features may not be readily apparent during plant growth, they have a crucial impact on overall plant performance, particularly for non-tuberous or rhizomatous crops (York et al., 2015). Root system architecture is closely related to a plant’s competitive advantage in the environment, including nutrient acquisition (Lynch, 1995; MansChadi et al., 2014), drought tolerance (Ribaut, 2006; Comas et al., 2013; Fenta et al., 2014; Wade et al., 2015), waterlogging tolerance (VanToai et al., 2001), and lodging resistance (Guingo et al., 1998).

In the field of seedling phenotypic analysis, seed viability testing, and seed germination experiments, parameters such as germination rate, seedling length, and growth rate are frequently measured. For instance, Wang Binbin et al. (Wang and Wu, 2022) conducted a study on the impact of extracellular polysaccharides from lactic acid bacteria on the germination and stress tolerance of japonica rice seeds. They performed statistical analysis on parameters such as germination potential, germination rate, root length, and shoot length of japonica rice seeds incubated in different culture solutions at a constant temperature for 7 days. However, this process required a significant amount of manual measurements. Similarly, Jiang Yuting et al. (Jiang et al., 2022) investigated the effects of different particle sizes and concentrations of polystyrene microplastics (PS-MPs) on the germination and seedling growth of sorghum seeds to understand the material’s impact on plants. These experiments also necessitated accurate measurements of germination, root length, and shoot length. Nevertheless, traditional manual measurement methods are no longer adequate to meet the demands of modern agriculture for efficient, precise, and automated measurements. Particularly in seed germination experiments, accurately measuring shoot length has become an urgent issue. Currently, there is a relatively limited body of research on methods for measuring shoot length during the seed germination stage, and there is no widely accepted automated detection method for measuring root or shoot length during seed germination.

In recent years, with the continuous progress of artificial intelligence, computer vision, and other technologies, more and more researchers have begun to explore how to utilize advanced technologies such as deep learning to solve problems in the field of agricultural detection. These studies have proposed a series of deep learning-based methods for image semantic segmentation and target detection to address the needs of modern agriculture. For example, Marset et al. (Marset et al., 2021) proposed a grape bud detection method based on the Fully Convolutional Network Mobile Network architecture (FCN-MN), which achieved improvements in segmentation, correspondence recognition, and localization, and realized the detection of the number of grape buds, bud area, and internode length. On the other hand, Yaying Shi et al. (Shi et al., 2022) achieved significant performance based on the YOLOv5 family of networks trained on a barley seed dataset, with the trained YOLOv5x6 model achieving a mean accuracy (mAP) of 97.5% in the recognition of barley seeds of different varieties. The development and application of these techniques provide new ideas and solutions to address automated seedling phenotyping, which is expected to play an important role in modern agriculture.

Considering the need for non-destructive, efficient, accurate, and consistent measurements for phenotyping rice seedlings, DeepLabv3+ (Chen et al., 2017) was used in this study as a baseline model for pixel-level segmentation of seedling images to extract the seedling’s shoot, radicle, and seed parts. Subsequently, the shoot and root lengths of the seeds were analyzed in depth by further length measurement analysis methods. In the field of image segmentation, the DeepLab family is one of the widely used and excellent models. DeepLabv3+ has achieved 89.0% and 82.1% test performance on PASCAL VOC 2012 and Cityscapes datasets, respectively (Chen et al., 2017), which is accurate enough for high-precision image segmentation tasks. However, the main backbone network of this model, Xception, has a large number of parameters, which consumes a significant amount of GPU memory. Additionally, the model’s memory footprint is substantial. As a result, it fails to meet the efficiency requirements for bud growth detection. To achieve fast and efficient detection, we optimized and improved the DeepLabv3+ model. We chose the FasterNet (Chen et al., 2023) network module with PConv as the backbone network to reduce the computational complexity. At the same time, we introduced the PSPA-ASPP structure and applied the EMA attention mechanism (Ouyang et al., 2023) to the network to improve the network operation speed and segmentation accuracy. This enables us to realize image segmentation in terms of efficiency and accuracy and significantly extends the applicability of the algorithm in practical applications. With this improvement, we can quickly and accurately recognize sprout root targets on the germination plate. After obtaining the target contour, we used a length recognition algorithm and performed skeleton extraction based on the sprout-root contour, thus obtaining a high-precision skeleton of the seed germination and realizing the automated detection of sprout length and root length.

The goal of this study is to perform detailed phenotyping of rice seed germination and seedling stages based on deep learning techniques and high-throughput plant phenotyping methods. By deeply investigating the phenotypic changes in these critical growth stages, we can better understand the mechanisms of plant growth, development, and adaptation to the environment, and provide strong support for plant breeding and crop improvement. Meanwhile, this study is also expected to reveal the dynamic changes in root system structure during plant seed germination and seedling growth, thus providing new strategies and directions for improving crop yield and adapting to planting under different environmental conditions.

The contributions or innovations of this paper are mainly the following:

(1) A deep-learning-based high-throughput phenotyping tool for hypocotyls is presented, which is fully automated and achieves the accuracy of a human expert in length measurement tasks across various plant species.
(2) Using a germination plate to simulate the growth environment of rice seeds, images of rice seedlings were collected under the germination plate. Three common phenotypic targets—shoots, roots, and seeds—were selected to produce the dataset.
(3) An efficient plant phenotype segmentation method is provided, which can achieve efficient segmentation of crop images at the pixel level.
(4) The FasterNet-DeepLabv3+ (DFMA) semantic segmentation model is proposed, which reduces the computational complexity of the network and the impact of hollow convolutional meshing. It improves detection efficiency and accuracy, and addresses the problem of frequent memory accesses and inefficiency caused by using depth-separable convolution in the original network.

## 2 Materials and methods

### 2.1 Image acquisition and data preparation

The dataset is divided into two parts. The first part is a homemade rice seedling dataset for training and testing the model. The second part is the publicly available dataset used to validate the generalization of the proposed model.

**Figure 1:**
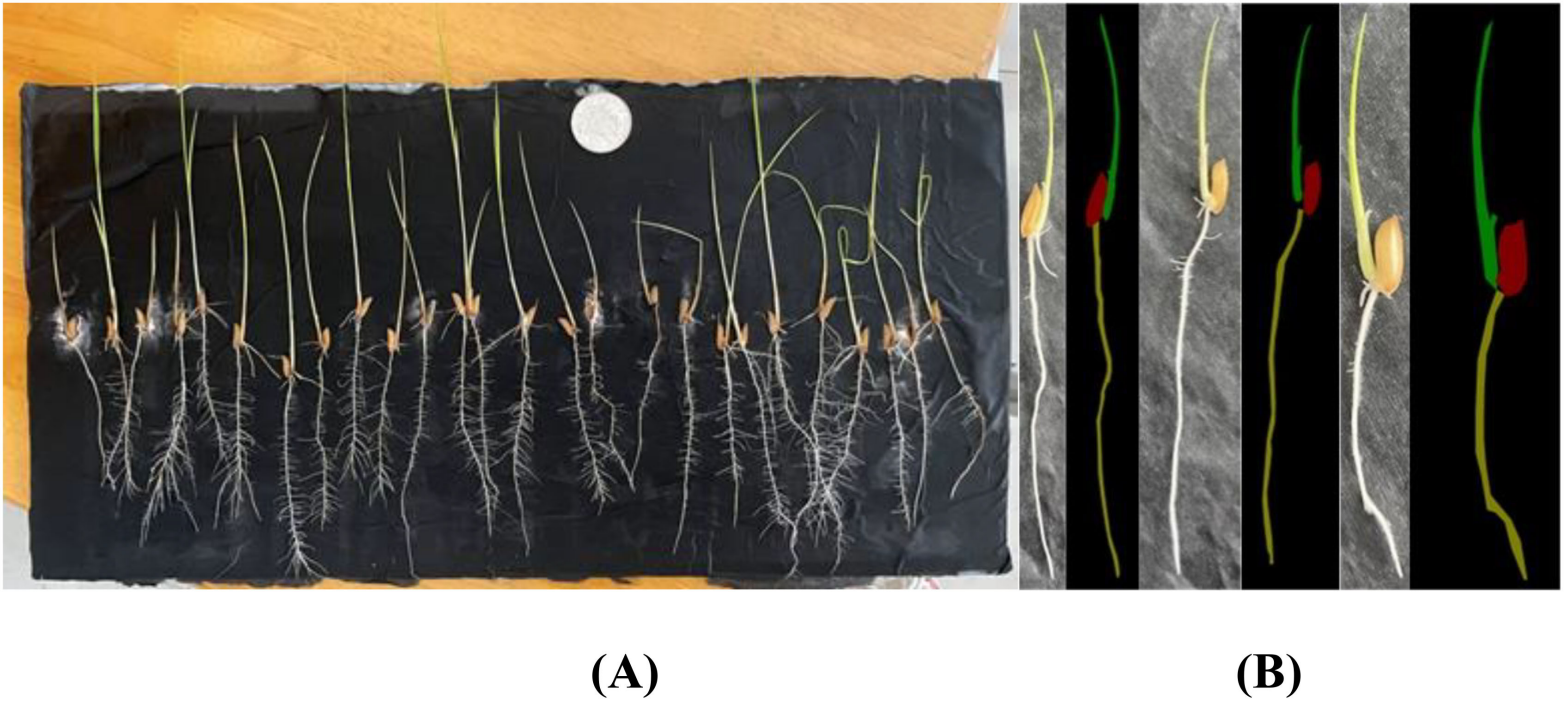
Homemade dataset germination plate pictures, **(A)** raw images, **(B)** mask images.

The construction of the self-made rice seedling dataset involves two main stages, beginning with the setup of the growth environment. To simulate the natural growth environment of rice and ensure sample consistency, a custom-designed germination board was developed for this experiment. Seeds were laid flat on a black velvet cloth, then gently clamped between two acrylic sheets, which secured both the cloth and seeds without disrupting the normal growth process or disturbing their stable positions. The germination board was placed vertically in an incubator set to a temperature of 28°C, thus controlling the temperature to provide optimal conditions for germination. To maintain a moist environment, water was evenly sprayed onto the seed surface every 12 hours using a spray bottle. This controlled environment minimized external disturbances, creating consistent experimental conditions. The experiment spanned the critical 7- to 14-day growth period for rice seedlings, during which there are significant morphological changes, from germination to the preliminary formation of plant structure, capturing key characteristics of each growth stage. Consequently, the dataset contains images of seedlings from various growth stages, establishing a foundational resource for model development to recognize growth stage characteristics. A germination board seedling image is illustrated in **Figure 1A**.

During the germination and image capture phase, the experiment ensured stable seedling growth on the germination board under constant temperature and humidity conditions. Images were taken using various mobile devices to increase dataset diversity. All images were captured perpendicular to the germination board to minimize viewpoint deviation, while the well-lit laboratory environment ensured high-quality image sources. The use of different devices introduced natural device noise, attributed to sensor variations or light reflections, enhancing both the dataset’s diversity and its robustness in real-world applications. To ensure data quality, all images were meticulously reviewed by botanical experts. A total of 115 healthy rice seedling samples were collected, spanning the 7- to 14-day growth period, thereby ensuring both representativeness and diversity in the dataset. In this study, Labelme open-source annotation software was used for manual image segmentation of images. The image was divided into four categories including shoots, roots, seeds and background. In the segmentation process, the parts of rice seedlings were separated from the background. For the fluff and secondary roots on the roots, they were treated as background. In this way, a homemade labeled dataset with the file suffix “.json” was obtained. Processed by the program, 115 sets of images were finally obtained. The sample image is shown in **Figure 1B**.

**Figure 2:**
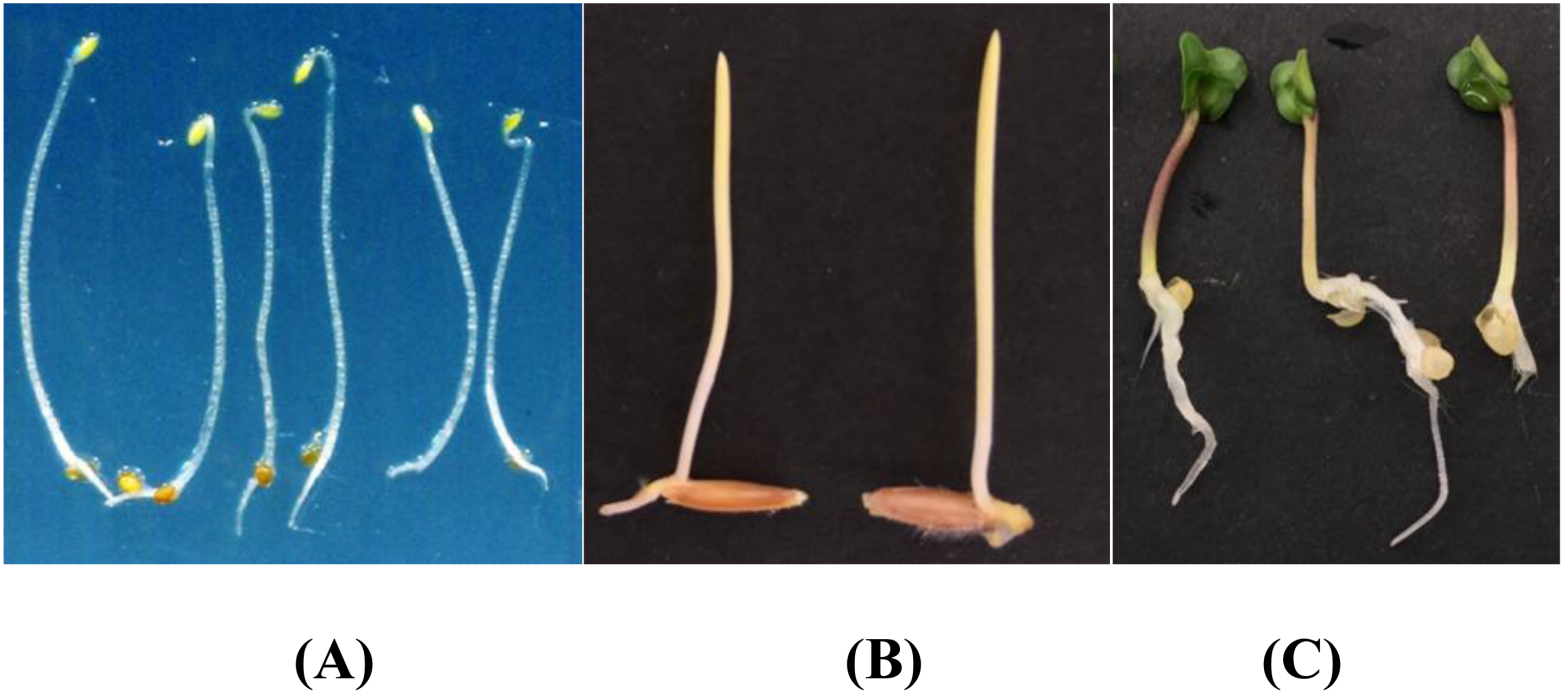
Plant segmentation public datasets. **(A)** Arabidopsis thaliana **(B)** Brachypodium dis-tachyon **(C)** Sinapis alba.

The public dataset was created using the Plant Segmentation Dataset, which was made public on the Kaggle platform by Orsolya Dobos et al. (https://www.kaggle.com/tivadardanka/plant-segmentation) in 2019. This dataset contains images of three seedlings, including *Arabidopsis thaliana*, *Brachypodium distachyon*, and *Sinapis alba*. The authors manually placed seedlings of these three plants on the surface of 1% agar plates and collected images using an EPSON PERFECTION V30 scanner. Images were saved in “.tif” or “.jpg” format using 800 dpi and 24-bit color settings. After collection, hypocotyls, cotyledons, seed coats, and roots were labeled using FIJI and used to create masks to train the segmentation algorithm. A sample of the dataset is shown in **Figure 2**.

### 2.2 Seedling phenotyping method

#### 2.2.1 FasterNet network model

Some common network models, such as MobileNet (Howard et al., 2017), ShuffleNet (Zhang et al., 2017), and GhostNet (Han et al., 2020), widely utilize Depth-wise Separable Convolution (DWConv) and Group Convolution (GConv) to extract spatial features. Depth-wise Separable Convolution is favored for its advantage in reducing the number of parameters. However, replacing 2D convolution with Depth-wise Separable Convolution may result in a drop in model performance, yielding suboptimal models. Furthermore, Depth-wise Separable Convolution places higher demands on memory access, leading to slower computation speeds on GPUs, lower FLOPs, and higher latency. Similarly, Group Convolution can reduce the number of parameters, but the limited interaction between channels within the group may result in the loss of global channel information. During the process of reducing parameters and FLOPs, the computational operators often experience the side effect of increased memory access. These networks are often accompanied by additional data operations, such as concatenation, shuffling, and pooling, and the runtime latency of these operations is crucial for small-scale models. The formula for calculating latency is as follows:


(1) $$\begin{array}{c} \text{Latency}=\frac{\text{FLOPs}}{\text{FLOPS}} \end{array}$$

One of them, FLOPS (floating point operations per second), is widely used to evaluate the effectiveness of computational speed. Although there are many approaches aimed at reducing FLOPs, few of them also consider low-latency optimization. To address this issue, the authors (Chen et al., 2023) introduced PConv and proposed FasterNet. as a new family of net-works with lower latency, on a variety of devices, FasterNet not only provides state-of-the-art performance, but also enables lower latency and higher throughput.

**Figure 3:**
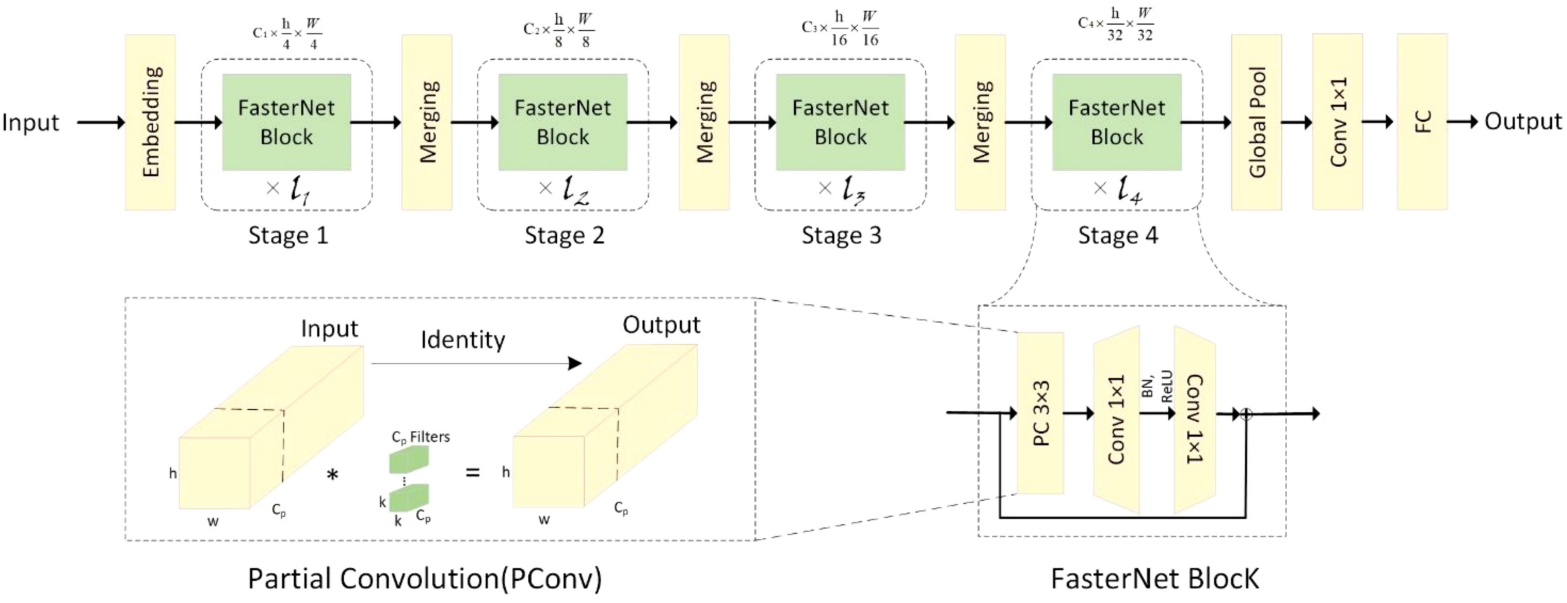
Overall architecture of FasterNet.

The overall architecture of FasterNet has four layers, each containing respectively l1, and l2, l3, and l4 individual FasterNet blocks, which are preceded by an embedding or merging layer. The last layer is used for feature classification. In each FasterNet block, there is one PConv and two PWConv layers, corresponding to the two Conv 1×1 layers shown in the bottom-right corner of **Figure 3**. The resulting feature maps are convolved 1×1 after data normalization and ReLU activation function to preserve the complexity of the feature maps and to achieve lower latency. where PConv is a convolution operator that reduces computational redundancy and memory access. **Figure 3**, bottom left, illustrates how PConv works. It simply applies regular Conv to a portion of the input channel for spatial feature extraction while keeping the rest of the channel unchanged. For consecutive or regular memory accesses, the first or last consecutive channel is computed by considering the first or last consecutive channel as a representation of the entire feature map. The input and output feature maps are considered to have the same number of channels without loss of generality. As a result, PConv reduces the FLOPs from $h\times w\times 2c^{\prime }+k^{2}\times c^{\prime }\approx h\times w\times 2c^{\prime }$ down to the number of channels in the $h\times w\times k^{2}\times c_{p}^{2}$ .

#### 2.2.2 EMA attention mechanisms module

Attention mechanism modules are employed in neural networks to improve the selection and integration of information from image data, thereby enhancing model performance and accuracy. Examples include SE (Squeeze-and-Excitation) (Hu et al., 2020), CBAM (Convolutional Block Attention Module) (Woo et al., 2018), and CA (Channel Attention) (Hou et al., 2021). The SE attention mechanism focuses solely on channel-level attention and is suitable for scenarios with a higher number of channels but performs poorly when channels are limited. CBAM requires more computational resources, increasing computational complexity and FLOPs. CA also incurs additional computational overhead as it computes attention weights for the entire feature map, and it cannot capture long-range dependencies.

**Figure 4:**
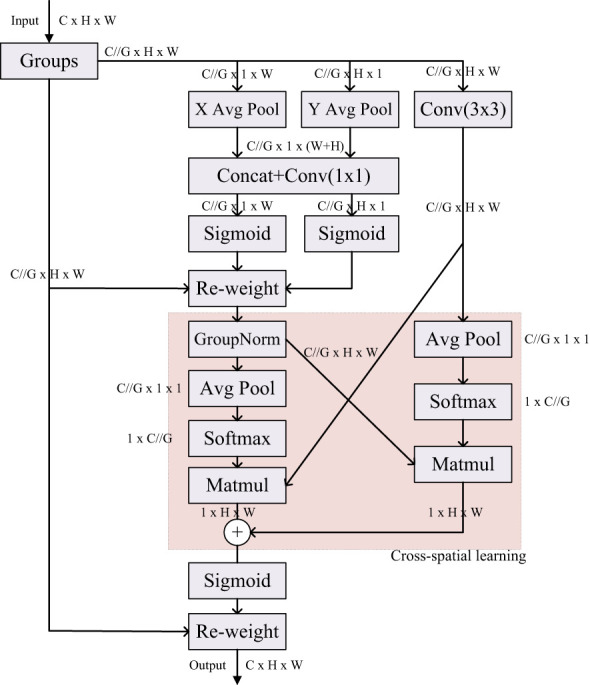
EMA self-attention module.

To further improve the performance of DeepLabv3+ network in extracting global information, we introduce a new efficient multi-scale attention module, EMA (Efficient Multiscale Attention) (Ouyang et al., 2023). EMA aims to preserve the information in each channel and reduce the computational overhead to achieve the goal of simultaneously preserving rich information and reducing the goal of computational cost. It achieves the effect of uniformly distributing spatial semantic features in each feature group by reconstructing some of the channels into batch dimensions and grouping the channel dimensions into multiple sub-features. The specific structure of EMA is shown in **Figure 4**.

A parallel substructure is used in the EMA module, which is applied in the attention mechanism to help the network avoid more parameters and greater depth, and the large local receptive fields of the neurons enable the neurons to collect multiscale spatial information. Therefore, EMA utilizes three parallel routes to extract the attention weight descriptors for the grouped feature maps. Two of the parallel routes are 1×1 branches and the third route is 3×3 branches. Cross-channel information interactions are also modeled in the channel direction. More specifically two 1D global average pooling operations are employed in the 1×1 branch to encode the channel along the two spatial directions respectively, while only one 3×3 kernel is stacked in the 3×3 branch for capturing multi-scale feature representations. Based on such a structure, EMA not only encodes the inter-channel information to adjust the importance of different channels, but also preserves the precise spatial structure information.

#### 2.2.3 PSPA-ASPP spatial pooling pyramid layer

**Figure 5:**
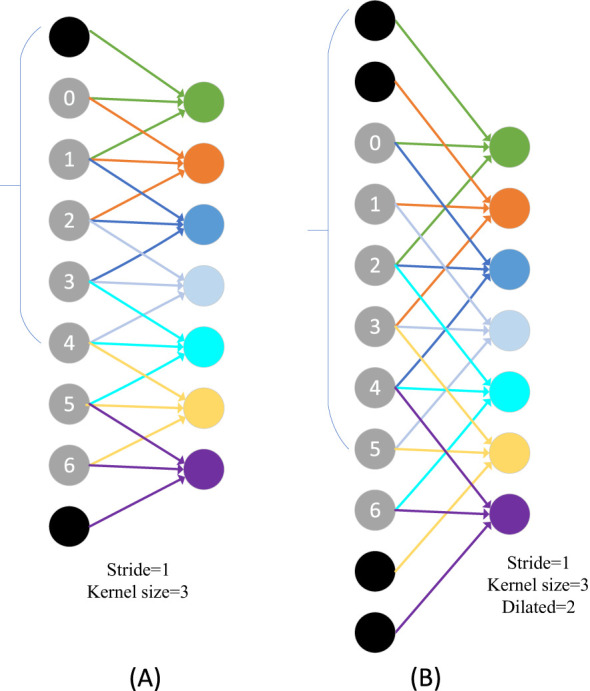
Visualization of the receptive field after the introduction of the null rate. **(A)** Represents regular convolution and **(B)** represents dilated convolution.

Inspired by Spatial Pyramid Pooling (SPP) (He et al., 2014), DeepLabv2 (Chen et al., 2017) introduced a novel module for semantic segmentation known as Atrous Spatial Pyramid Pooling (ASPP). The ASPP module’s design is primarily based on the concept of dilated convolution. Traditional image segmentation algorithms often use pooling and convolution layers to increase the receptive field while simultaneously reducing the feature map size. However, when it becomes necessary to upsample or restore the size of feature maps from downsample and pooled layers, it can lead to a loss in the accuracy of image features and potential loss of semantic information from the original image. To address this issue, a method is needed that can increase the receptive field while keeping the feature map size unchanged, thus replacing upsampling and downsampling operations. Dilated convolution is precisely designed to meet this requirement. Dilated convolution extends the receptive field of convolutional operations by introducing holes (gaps) in the convolution kernel without changing the kernel’s size. Specifically, dilated convolution introduces some virtual zero-value pixels in the convolution operation, allowing the expansion of the convolution kernel’s receptive field without altering the feature map size. **Figure 5A** represents regular convolution, while (**Figure 5B**) represents dilated convolution with a dilation rate of 2, providing a comparison of the changes in receptive field between the two. ASPP’s design represents a typical application of dilated convolution, achieving multiscale target information by parallelizing three dilated convolutions with different dilation rates, along with a standard convolution and a pooling operation.

**Figure 6:**
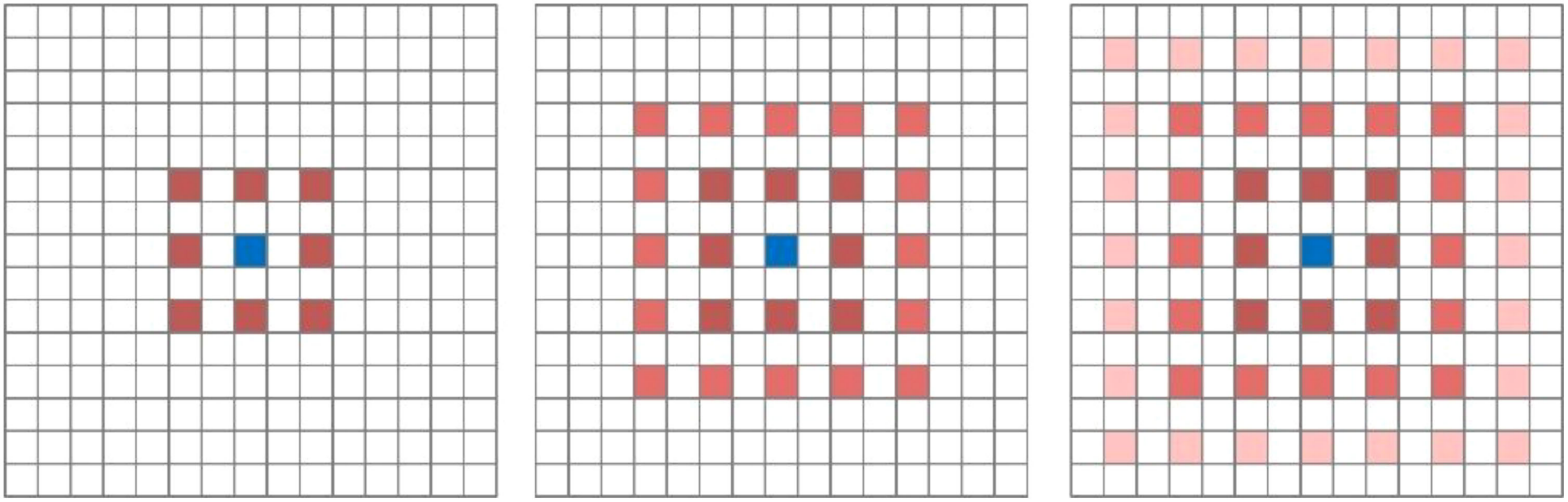
Mapping of gridding effects. From left to right, the dilation rates are 2, 2, and 2, respectively. Following the approach outlined by Shi et al (Shi and Bao, 2023), our research team devised a novel ASPP (Atrous Spatial Pyramid Pooling) structure known as PSPA-ASPP. Firstly, we replaced the original ASPP’s first branch layer’s 1×1 convolution with a 3×3 Pconv convolution to broaden the receptive field of the first layer while avoiding redundant learning. Secondly, we employed two 3×3 dilated convolutions with dilation rates of 2 and 3, each with 128 convolution kernels, which is half of the original ASPP’s individual branch, and concatenated them in the channel dimension. Subsequently, we applied two additional 3×3 dilated convolutions with dilation rates of 5 and 7 in a similar concatenated manner. This design allows the network to capture features from different scales while substantially reducing the grid effect and making more effective use of feature layer information. The final layer still employs average pooling to capture global features of the feature map. **Figure 7** illustrates the overall network architecture of PSPA-ASPP.

Although introducing dilated convolutions can increase the receptive field, it also suffers from two significant drawbacks. Firstly, dilated convolutions can lead to the problem of sparse sampling. While dilated convolutions excel in extracting global information, they may lack some semantic information when dealing with small targets. This is because larger dilation rates can result in excessive gaps between sampled points, making it challenging to capture fine details of small objects. Secondly, dilated convolutions exhibit the grid effect issue. When the same dilation rate is used or there exists a common divisor greater than 1, during the process of feature map stacking, it may lead to the loss of local detailed information in image features, resulting in a pixelated grid-like effect in the im-ages. This occurs because the same dilation rate or common divisor causes multiple sampled points to form a regular grid structure on the feature map, preventing the recovery of certain local information. **Figure 6** illustrates the gridding effect of feature maps. When three consecutive convolution operations with a dilation rate of 2 and a kernel size of 3×3 are applied to a feature map, not all pixels on the feature map participate in the computation.

**Figure 7:**
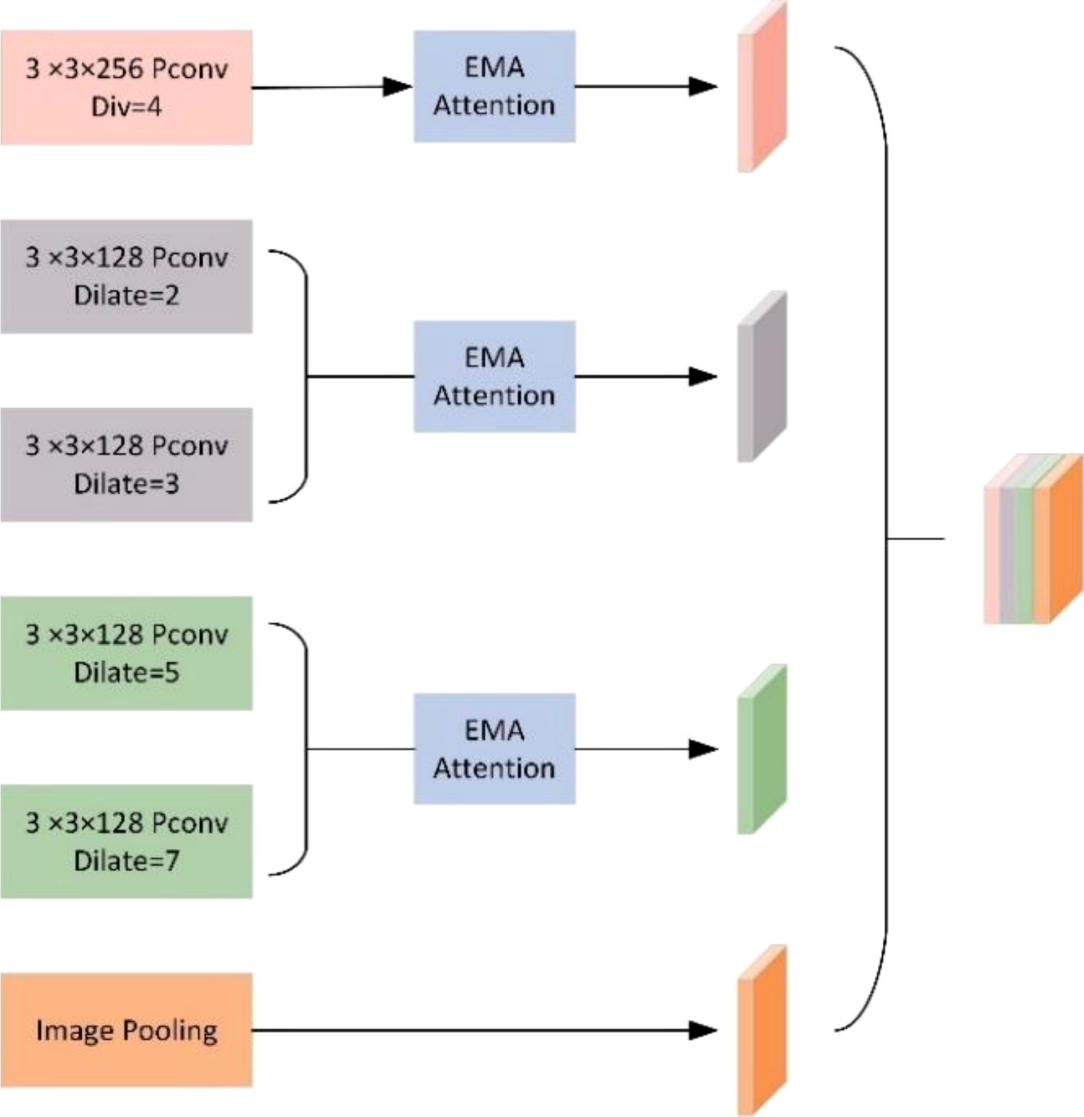
PSPA-ASPP series-parallel network structure diagram.

#### 2.2.4 CARAFE up-sampling operator

The operator for feature upsampling is essential for increasing the resolution of low-resolution feature maps to match the size of high-resolution feature maps, and the design of an effective upsampling operator is of paramount importance (Mazzini, 2018; Chen et al., 2021; Dai et al., 2021). Among the widely used feature upsampling operators, nearest-neighbor interpolation and bilinear interpolation only consider sub-pixel neighborhoods, failing to capture the rich semantic information required for dense prediction tasks. The Transposed Convolution (Dumoulin and Visin, 2016), serving as the inverse operator of convolutional layers, employs convolution kernels of the same size throughout the entire image, thereby neglecting local information variations and leading to a significant increase in parameter count.

Wang et al. (Wang et al., 2019) introduced the CARAFE (Content-Aware ReAssembly of Features) feature re-sampling operator, which adaptively aggregates information within larger receptive fields, while maintaining remarkable computational efficiency. CARAFE generates weights in a content-aware manner by combining features within predefined regions near the central position. Multiple sets of such upsampling weights are computed for each central position, and the resulting features are rearranged into spatial blocks to complete the feature upsampling process. To validate the effectiveness of the CARAFE operator, the original authors conducted extensive experiments on Faster RCNN (Ren et al., 2015), employing various operators for upsampling within the Feature Pyramid Network (FPN). The results, as shown in **Table 1**, included cases denoted as “nearest neighbor + convolution” (N.C.) and “bilinear + convolution” (B.C.), where an additional 3×3 convolution layer was added after the corresponding upsampling. The comparative experiments also included three typical upsampling methods: deconvolution (Deconv), pixel shuffle (P.S.), and guided upsampling (GUM), as well as spatial attention (S.A.). CARAFE exhibited the highest average precision (AP) among all upsampling operators while maintaining lower FLOPs and parameter counts, indicating its efficiency in enhancing detail recovery and excelling in model lightweighting. Results for N.C. and B.C. suggested that additional parameters did not yield significant gains, whereas Deconv, P.S., GUM, and S.A. all exhibited inferior performance compared to CARAFE.

**Table 1:** Comparison of the performance of sampling operators on CARAFE.

Method	AP	FLOPs	Params
Nearest	36.5	0	0
Bilinear	36.7	8k	0
N.C	36.6	4.7M	590K
B.C	36.6	4.7M	590K
Deconv	36.4	1.2M	590K
P.S	36.5	4.7M	2.4M
GUM	36.9	1.1M	132K
S.A	36.9	28K	2.3K
CARAFE	37.8	199K	74K

**Figure 8:**
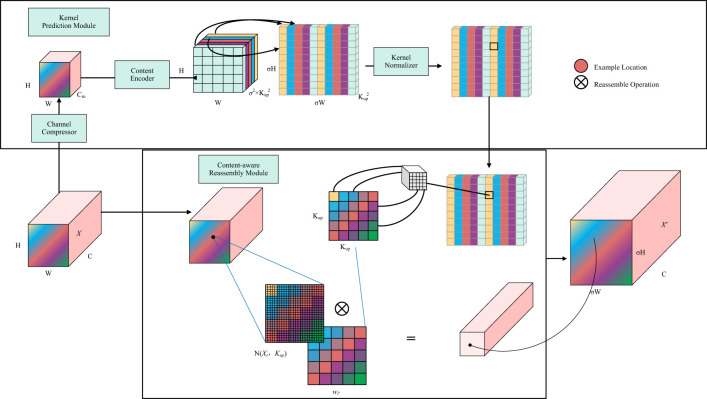
CARAFE up-sampling operator.

As shown in **Figure 8**, CARAFE, as an upsampling operator with a content-aware kernel, consists of two steps. The first step is to predict the reassembly kernel for each target position based on its content (i.e., the Kernel Prediction Module in **Figure 8**). The second step is to use the predicted kernel to reassemble the features (i.e., the Content-aware Reassembly Module in **Figure 8**). In the first step, a feature map X of size C×W×H is upsampled by a factor of σ, resulting in a new feature map of size C×σH×σW. Assuming an upsample kernel size of $k_{up}\times k_{up}$ , if different upsample kernels are desired for each position in the output feature map, the predicted upsample kernel should have a shape of $\sigma H\times \sigma W\times k_{up}\times k_{up}$ . To compress the input feature map, a convolution layer with a kernel size of $\;k_{encoder}\times k_{encoder}$ is used to predict the upsample kernel, with an input channel number of $C_{m}$ and an output channel number of $\sigma^{2}k_{up}^{2}$ , resulting in an upsample kernel of shape $\sigma H\times \sigma W\times k_{up}^{2}$ . In the second step, for each position in the output feature map, it is mapped back to the input feature map, and a $k_{up}\times k_{up}$ region centered on that point is extracted. The dot product is then computed between the extracted region and the predicted upsample kernel for that point to obtain the output value. Different channels at the same position share the same upsample kernel.

In the improved Deeplab v3+ network, as illustrated in Equation 2, the kernel prediction module ψ predicts the position for each location based on the learned weights $\;W_{l^{\prime }}$ in the first step. Subsequently, as described in Equation 3, the content-aware recombination module ϕ recombines the features $X_{l}$ with the kernel $W_{l^{\prime }}$ in the second step. To reduce the parameter count of upsampling operators and enhance efficiency, an 8-fold upsampling CARAFE module is introduced after the ASPP module, which restores the size of the feature maps from 256 × 16 × 16 to 256 × 128 × 128. Following feature fusion, a 4-fold upsampling operation is applied to restore the final feature map to 4 × 512 × 512 dimensions.


(2) $$\begin{array}{c} W_{{\text{l}}^{\prime }}=\text{ψ}\left(\text{N}\left(X_{\text{l}},{\text{k}}_{\text{encoder}}\right)\right) \end{array}$$

(3) $$\begin{array}{c} X_{{\text{l}}^{\prime }}=\text{ϕ}\left(\text{N}\left(X_{\text{l}},{\text{k}}_{\text{up}}\right),W_{{\text{l}}^{\prime }}\right) \end{array}$$

#### 2.2.5 DFMA overall network structure

The DFMA model integrates the FasterNet backbone with the SPA-ASPP module enhanced by an EMA attention mechanism, aimed at improving feature extraction and segmentation accuracy for plant seedling images while being optimized for mobile deployment. Initially, the input RGB image undergoes feature extraction via the FasterNet backbone. FasterNet leverages a hybrid structure combining Pconv, PWconv, and standard convolution to efficiently extract both low-level and high-level features, overcoming the limitations of depthwise separable convolution. To ensure the participation of shallow features in subsequent processing, the model retains shallow feature maps downsampled four times within the backbone network. Following this, DFMA introduces an EMA (attention mechanism) module that enhances the fusion capability of high-level features. The EMA mechanism dynamically reweights features from different layers, enabling the network to focus on key parts of the image when extracting high-level features, thus boosting overall performance.

During the multi-scale feature extraction stage, DFMA employs the SPA-ASPP module with EMA attention. This module captures high-level semantic information across multiple scales through several branches, effectively avoiding grid effects common in traditional methods. The EMA attention mechanism further strengthens the representation capacity of these branches, allowing the model to concentrate on crucial features within plant seedling images.

**Figure 9:**
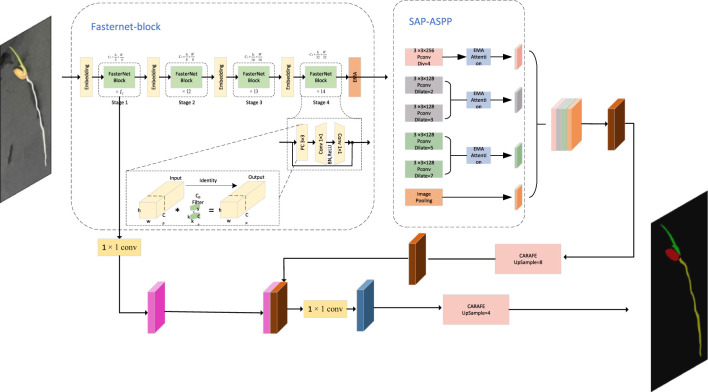
Improvement of the overall architecture of FasterNet-Deeplab v3+.

In the decoding stage, the multi-scale feature information is merged and upsampled using the CARAFER operator, aligning the high-level feature map dimensions with the low-level feature map for subsequent fusion. DFMA applies a 1×1 convolution on the shallow feature map to match channel dimensions with the upsampled deep feature map, preparing it for concatenation. The concatenated feature map then undergoes partial convolution and additional upsampling, ultimately generating the model’s prediction. This integrated design combines the strengths of FasterNet and the SPA-ASPP module, enhancing the model’s feature extraction capacity while ensuring efficiency and accuracy for mobile deployment. The DFMA model structure is shown in **Figure 9**.

### 2.3 Seedling length detection method

Through training the DFMA network model, we can easily input seedling images for analysis and obtain corresponding masks. These masks accurately represent the different positions of seeds within the images, allowing researchers to observe the developmental details of seed germination, embryonic axis, and root structure clearly. In certain studies, it is not only necessary to conduct in-depth analysis of the development of various plant parts but also to acquire precise parameters for these developmental aspects. Therefore, we introduce a seedling length measurement algorithm, which not only provides accurate segmentation masks for the images but also enables us to obtain exact parameters for the development of different plant parts.

In this seedling length detection, we divided into two main steps. First, we skeletonize the image using the Hilditch algorithm to obtain the median length of the segmented image. Secondly, we utilize Hough Transform to obtain the transformation relationship between the true length of the seedling detection site and the pixels.

The Hough Transform is an early image processing algorithm that employs a voting-based approach for shape fitting. Its objective is to mathematically describe certain edges in an image to enhance information extraction. Unlike alternative techniques such as least squares, robust estimation, and RANSAC, the Hough Transform excels in simultaneously fitting multiple objects. The detection process in the Hough Transform involves iterating through all non-zero points, accumulating votes for each point’s center, and assigning scores. For each point along a circle, its center lies on the vector perpendicular to the point and passing through the point’s location. The intersection point of these center vectors corresponds to the desired circle center position. In this experiment, coins serve as a real-world scale for converting lengths to pixels, enabling the detection of coin diameters. Within the Hough Transform, fitting circles requires three parameters - (x,y,r) , where x and y denote coordinates, and r represents the circle’s radius. These parameters are determined using the following formula:


(4) $$\begin{array}{c} \left(\text{X}-{\text{x}}^{2}\right)+\left(\text{Y}-{\text{y}}^{2}\right)={\text{r}}^{2} \end{array}$$

**Figure 10:**
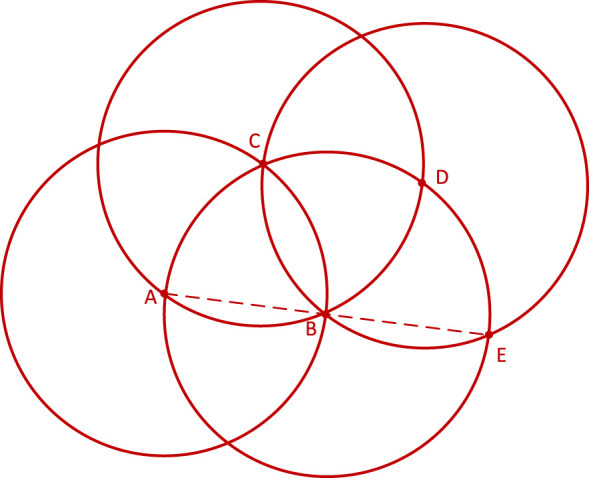
Hough gradient method.

The Hough Gradient method optimizes the standard Hough Circle Transform by eliminating the need to draw complete circles in parameter space for voting. Instead, it calculates the gradient vectors at contour points and casts votes along the gradient direction, at a distance of R in both directions from the contour point, effectively conducting one vote on each side. Ultimately, the circle center’s position is determined based on the voting results as depicted in **Figure 10**.

As shown in the diagram, assuming that the gradient directions of the contour points ACDE all pass through point B, they will each cast a vote for point B. Within a search radius of R, votes are cast on both sides of the contour points at a distance of R based on the gradient direction. Ultimately, the center position is determined based on the voting results. Compared to the parameter space voting method for determining the center, this approach offers better resistance to interference. Even if other points also cast votes, their voting results are too dispersed, and their interference with the overall voting result can be almost negligible.

For this experiment we use coins as a scale between real and pixel values, and the actual value of the sprout length can be calculated based on the coin diameter. A dollar coin as a circle with a diameter of 25mm, get how many pixels it occupies in the figure, it can get the number of pixels per metric (pixel Per Metric), and then calculate the pixels occupied by other objects n, it can get the actual length (n × pixel Per Metric).

## 3 Experiments and results

### 3.1 Model evaluation criteria

In this network model of bud root region segmentation, the deep learning network mainly adopts Mean Intersection over Union (*mIoU*) as the evaluation index of the model, and mean intersection over union refers to the ratio of intersection and concatenation values between the true and predicted values of each classification, and then averages over multiple classifications.

In the field of scientific research and data analysis, True Positive (*TP*)is defined as the portion where both the actual value and the predicted value are true. True Negative (*TN*)corresponds to cases where both the actual value and the predicted value are false. False Positive (*FP*)refers to instances where the actual value is false, but the predicted value is true. False Negative (*FN*) denotes situations where the actual value is true, but the predicted value is false.


(5) $$\begin{array}{c} \text{MIoU}=\frac{1}{\text{k}+1}\sum_{\text{i}=0}^{\text{k}}\frac{\text{TP}}{\text{FN}+\text{FP}+\text{TP}} \end{array}$$

In addition to mIoU, precision (*Pre*), recall (*Rec*), and accuracy (*Acc*) are also used as evaluation metrics for the algorithm. Precision (*Pre*) is used to measure the proportion of predictions that are correct in the samples that the model predicts as positive examples, with the formula shown in Equation 6:


(6) $$\begin{array}{c} \text{precision}\;=\frac{\text{TP}}{\;\text{TP}+\text{FP}} \end{array}$$

Recall (*Rec*) is the proportion of all positive cases that the model predicts correctly, as shown in Equation 7:


(7) $$\begin{array}{c} \text{recall}\;=\frac{\text{TP}}{\;\text{TP}+\text{FN}} \end{array}$$

Accuracy (*Acc*) is the number of samples with all correct predictions as a percentage of all samples. The higher its value, the better the model. As shown in Equation 8:


(8) $$\begin{array}{c} \text{Accuracy}\;=\frac{\text{TP}+\text{TN}}{\text{TP}+\text{TN}+\text{FP}+\text{FN}} \end{array}$$

### 3.2 Data augmentation settings in the training phase

In this study, we employed online data augmentation techniques to enhance the robustness and generalization capability of the model. The data augmentation operations included random scaling (with a scale range of 0.25 to 2 times), aspect ratio distortion, horizontal flipping (with a probability of 50%), gray padding (pixel value of 128), random adjustments to hue, saturation, and brightness in the HSV color space, as well as random cropping and shifting. These augmentation methods were dynamically applied to the training data’s images and labels during each training iteration, thereby expanding the original data distribution, simulating target variations under different scenarios and conditions, and significantly improving the model’s adaptability to changes in lighting, orientation, and target shapes. Moreover, dynamic augmentation reduced the need for storing pre-augmented data while significantly increasing data diversity, thereby improving training effectiveness. It is important to note that data augmentation was only applied during the training phase and not during the validation phase to ensure that the validation results objectively reflect the true performance of the model. The experimental results demonstrate the effectiveness of the proposed method in reducing overfitting and improving model performance.

### 3.3 Experimental platform and parameter design

The network is implemented based on the PyTorch library and trained on a single Nvidia RTX 3060 GPU, with a 12th Gen Intel(R) Core(TM) i5 - 12400F processor. The initial batch size is set to 10, and the initial learning rate is 0.05. Stochastic Gradient Descent (SGD) is adopted as the optimization method, and both Dice loss and cross - entropy loss are utilized as the objective functions. L2 regularization is applied for model regularization. We use online data augmentation techniques, such as rotation (by 90, 180, and 270 degrees), horizontal flipping, and random adjustments to hue, saturation, and brightness in the HSV color space. The original dataset contains 115 images, which are split into a training set of 92 images and a validation set of 23 images following an 8:2 ratio. Through these online augmentation operations, each original training image can generate multiple variants during each training iteration. To estimate the approximate quantity of the augmented training data, considering that each image has 7 different augmented forms on average (3 rotations + 1 horizontal flip + 3 color space adjustments), the total number of augmented training images is about 644. During training, the batch size is adjusted to 8. The training process will automatically stop when the loss function output of the validation set does not decrease for 20 consecutive epochs, with a maximum of 500 epochs permitted. The segmentation performance is evaluated on the validation set using the Mean Intersection over Union (mIoU) metric (**Table 2**).

**Table 2:** Training parameters.

Parameter	Value
Initial learning rate	0.005
End Lr	0.0001
Momentum	0.937
Batch size	8
Lr policy	Adam
Lr decay	cos
epoch	500

### 3.4 Evaluation of the results of the seedling phenotype segmentation experiment

According to the analysis results in **Table 3**, it is evident that FasterNet exhibits significant advantages in network backbone selection. Moreover, during the experimental phase, we observed that FasterNet’s training process is notably faster, which may be attributed to the frequent memory access associated with depth-wise separable convolutions and pointwise convolutions used in Xception and MobileNet. In our proposed PSPA-ASPP structure, when the backbone networks are the same, the combination of FasterNet with ASPP achieves an mIoU of 79.84, whereas when combined with PSPA-ASPP, it reaches 81.36. It is noteworthy that FasterNet+PSPA-ASPP also boasts lower GFLOPs, indicating its competitiveness in terms of computational efficiency. The final experimental results demonstrate that the FasterNet+EMA+PSPA-ASPP+CARAFE combination exhibits the best performance, further substantiating its outstanding performance in image segmentation tasks.

**Table 3:** Results of ablation experiments.

Xception	MobileNetV2	FasterNet	CA	SP	EMA	PSPA_ASPP	CARAFE	MIoU/%	GFLOPs/G
✔								67.09	167.00
	✔							74.21	53.03
		✔						79.84	138.70
		✔	✔					78.79	138.71
		✔	✔	✔				81.32	141.52
		✔		✔	✔			81.35	139.45
		✔				✔		81.36	135.23
		✔			✔		✔	81.63	139.83
		✔			✔	✔		81.58	137.29
		✔			✔	✔	✔	**81.72**	137.67

Bold value represents the highest mIoU achieved by our model in the tests.

The primary objective of this experiment is to achieve more precise phenotypic analysis; therefore, when differences in other metrics are minimal, this study prioritizes model accuracy. The improved FasterNet-DeepLab V3+ achieves the highest mIoU while significantly reducing GFLOPs. By simplifying the branches with the PSPA-ASPP module, the GFLOPs are reduced by approximately 2.161 G, effectively enhancing the model’s learning capacity.

**Figure 11:**
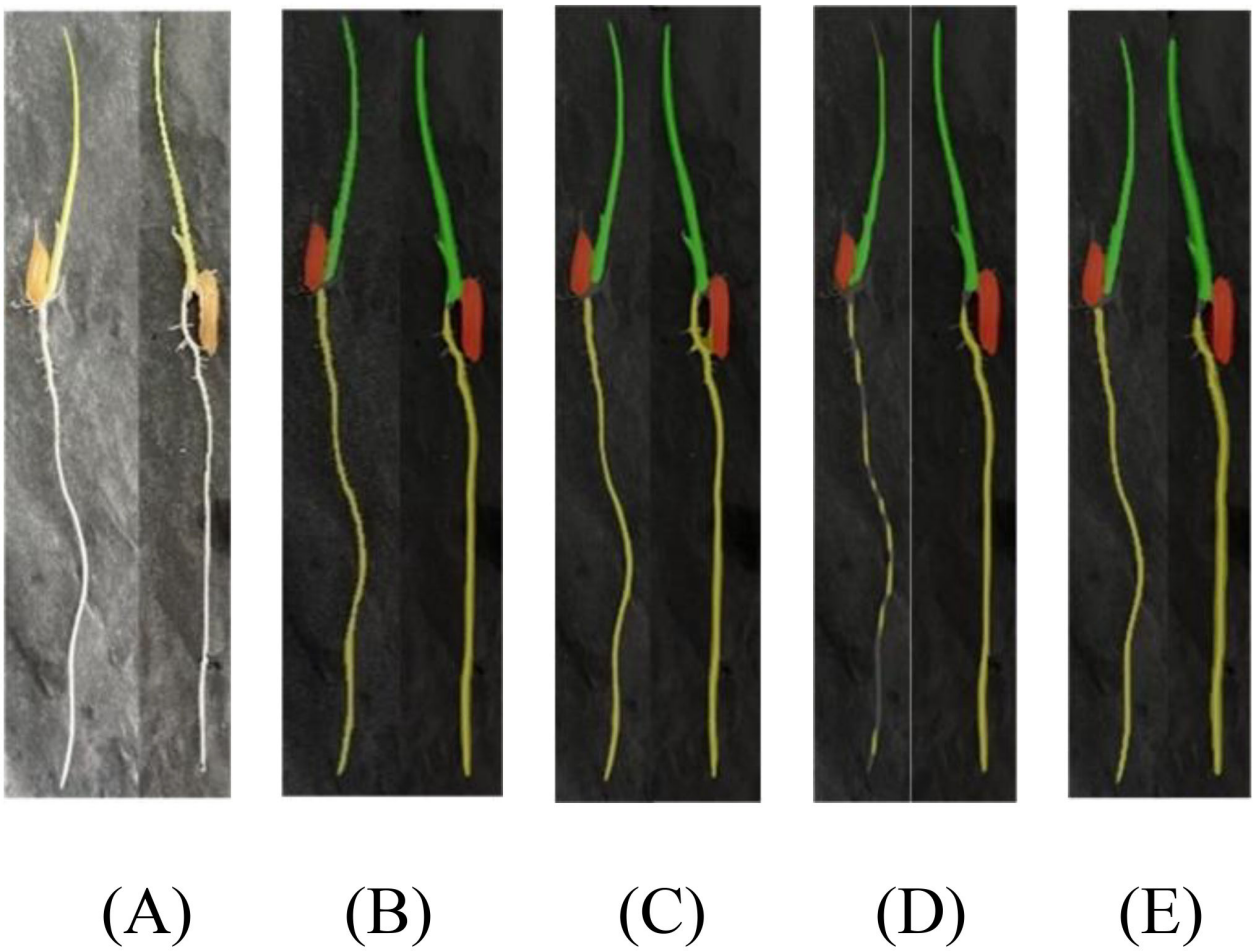
Phenotypic recognition results for homemade datasets. **(A)** Original figure. **(B)** DFMA. **(C)** deepLab v3+-MobileNet. **(D)** deepLab v3+-Xception. **(E)** UNet-VGG.

In accordance with **Figure 11**, we conducted a comparative experimental analysis of prediction results using the DeepLabv3+ semantic segmentation model with MobileNet and Xception as backbone networks, the Unet-VGG segmentation model, and our improved DFMA network in our research. As evident from the results in **Figure 11D**, the segmentation performance is the poorest in this case, with issues of coherence in the regions covered by masks for rice seedling shoots and root areas, resulting in suboptimal segmentation. In contrast, our proposed DFMA network model exhibits the best performance, accurately segmenting each region.

On the public dataset, the DFMA was compared with networks such as UNet (a network provided by the original authors of the public dataset), MobileNetV2, and Xception in terms of equalization and concurrency results, as shown in the **Table 4**.

**Table 4:** MIoU results (%) of different network trainings on public dataset.

Model	Brachypodium distachyon	Sinapis alba	Arabidopsis thaliana
DFMA	87.69	91.07	66.44
MobileNetV2	84.84	87.21	63.39
Xception	78.78	68.75	56.22
UNet-VGG	80.10	85.65	62.82

**Figure 12:**
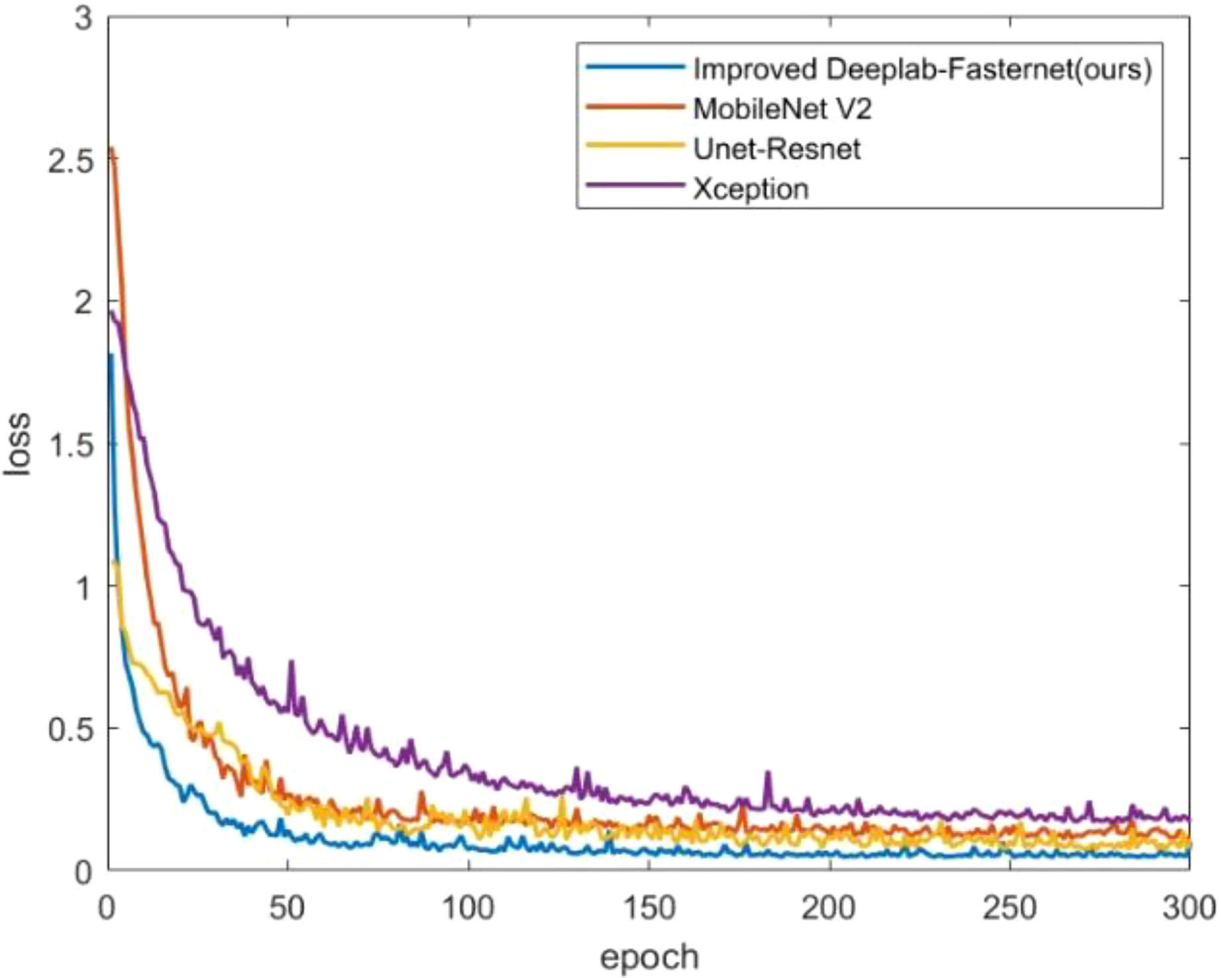
Loss curves of different models on two-spike phragmites picture dataset.

Based on the analysis results presented in **Table 4** and illustrated in **Figure 12**, it is evident that our proposed DFMA model demonstrates exceptional performance on publicly available datasets, outperforming other models. Across three distinct plant datasets, namely short-stalked grass, white Sinapis alba, and Arabidopsis thaliana, the DFMA model achieves average intersection over union (mIoU) ratios of 87.69%, 91.07%, and 66.44%, respectively, surpassing the other two models by at least 2 percentage points. Furthermore, as depicted in **Figure 12**, during the training process, it is apparent that the DFMA network model converges more swiftly and maintains a lower loss function value, providing additional evidence of its superior performance and efficiency.

In accordance with **Table 5**, our proposed DFMA network achieves the best segmentation performance on publicly available datasets. Due to the limitations of depth-wise separable convolution, the MobileNet network exhibits poor mask recognition in the bud apex region. Conversely, due to its restricted network depth, UNet produces relatively coarse results in fine detail recognition.

**Table 5:** Plant phenotype segmentation results for different networks of the open dataset.

	original figure	Improvement of FasterNet-Deeplab V3+	Deeplab V3+-MobileNet	UNet-VGG
Arabidopsis thaliana	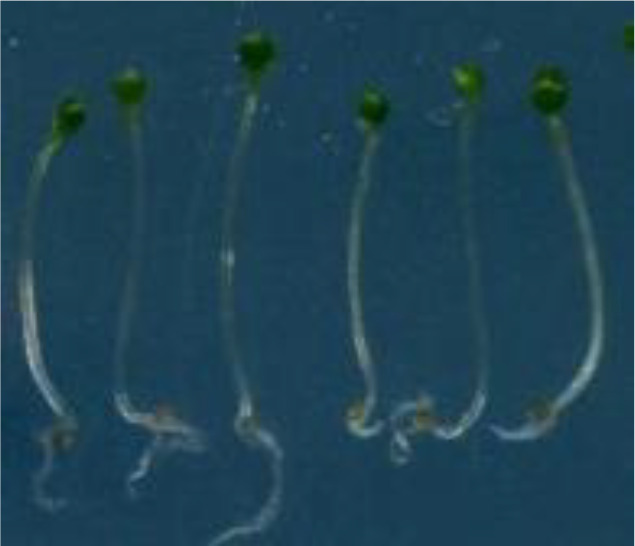	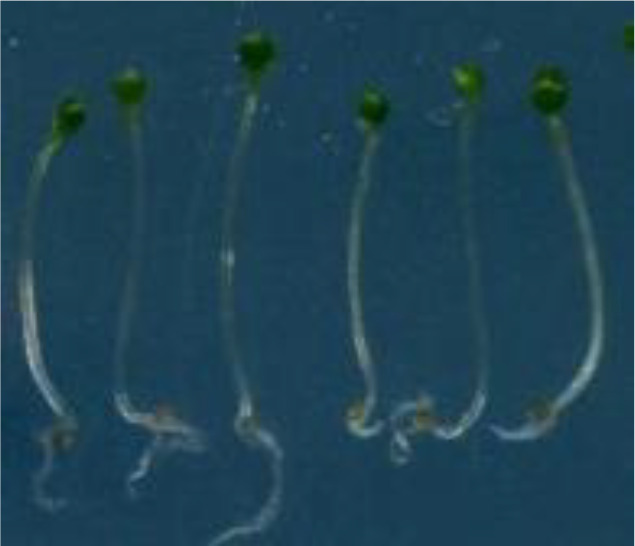	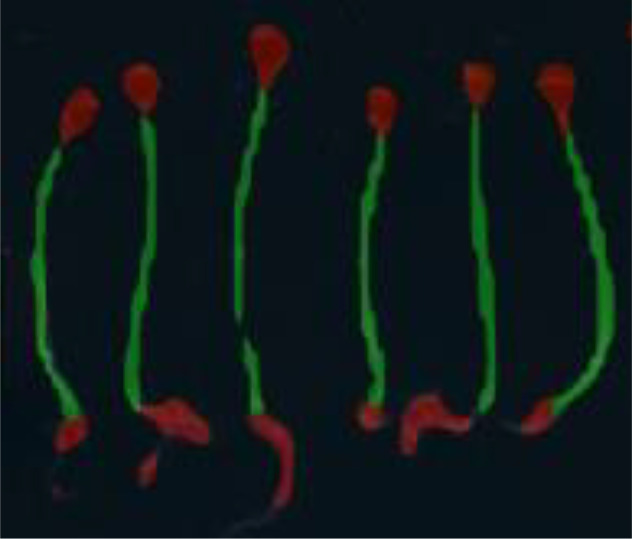	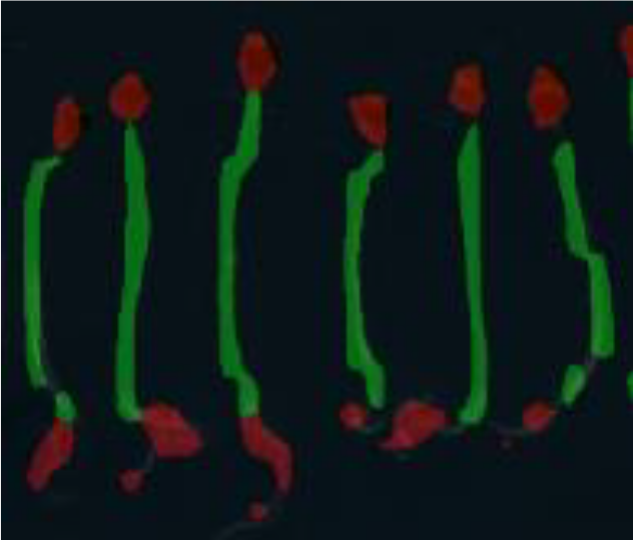
Brachypodium distachyon	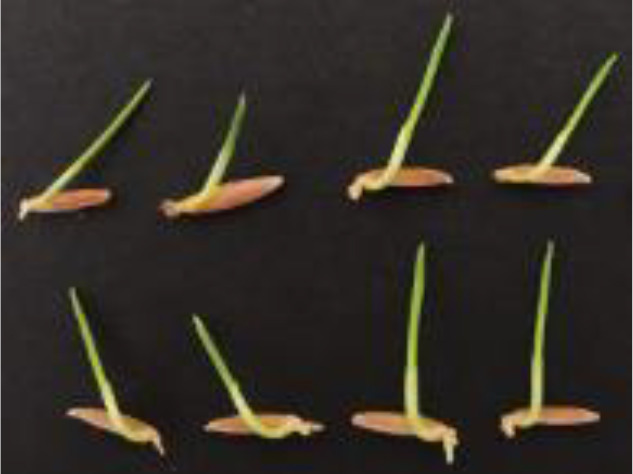	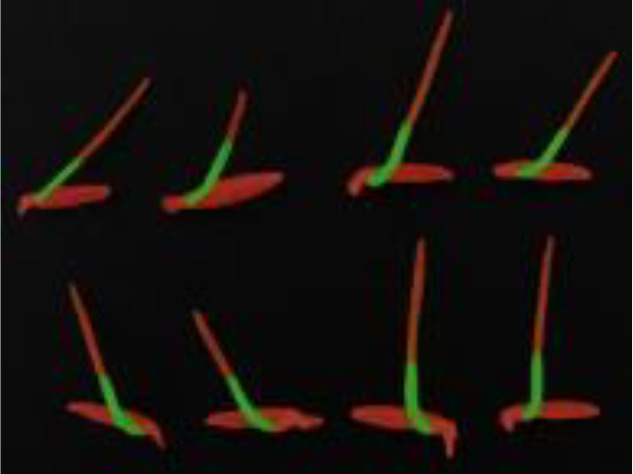	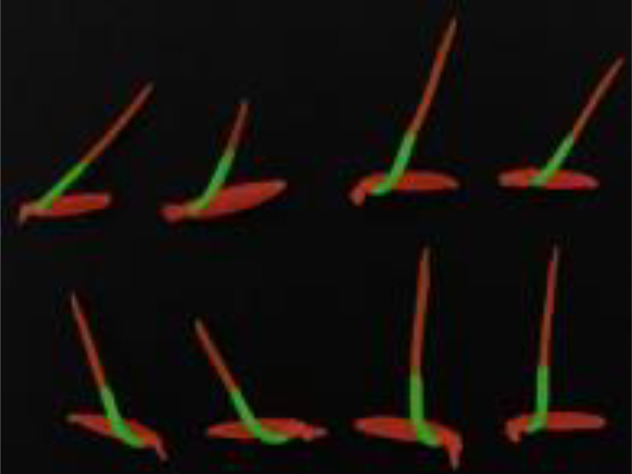	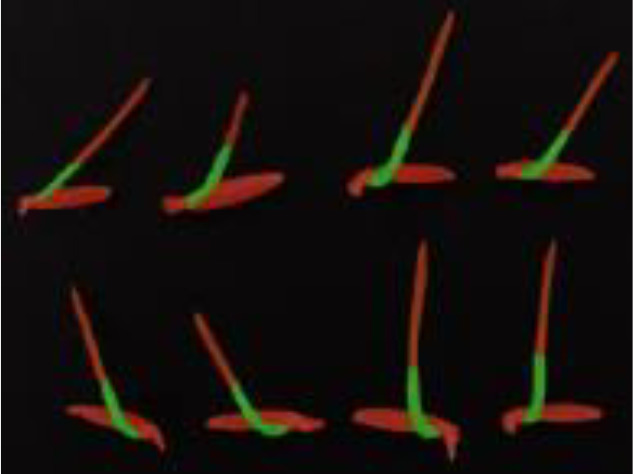
Sinapis alba	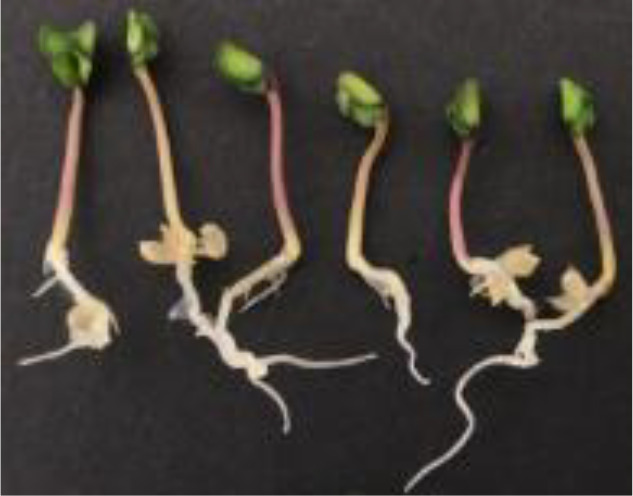	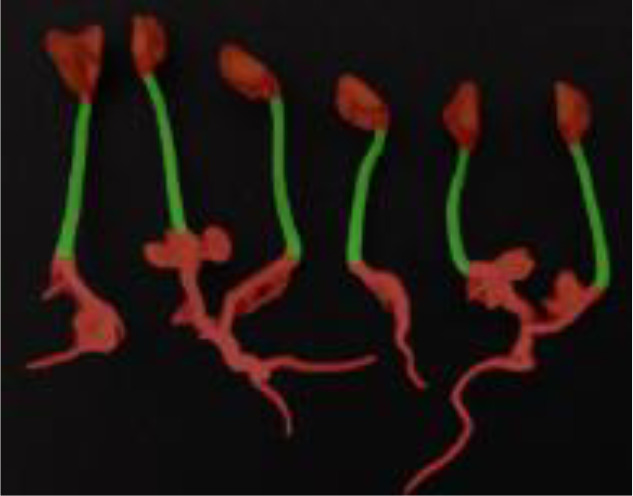	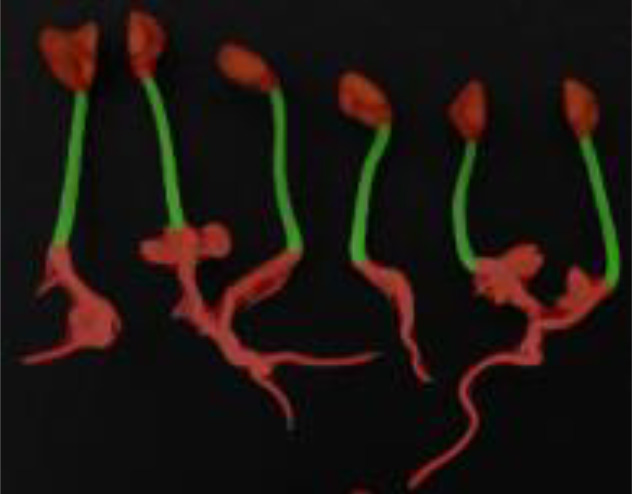	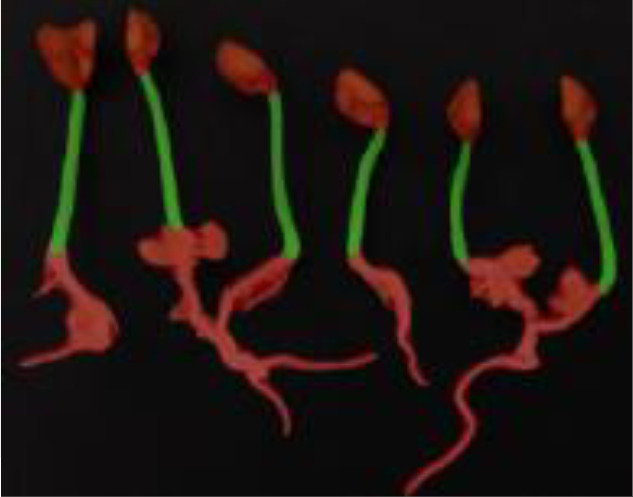

The DFMA model outperforms other models in plant phenotyping analysis, largely due to its design tailored to address the unique challenges of seedling segmentation tasks. Plant phenotyping often requires accurate identification of intricate and complex structures. Seedling images commonly contain multi-scale, fine structural features, such as leaf edges and stems, which demand high segmentation precision. Additionally, the execution environment for seedling segmentation tasks is typically resource-limited, such as mobile devices or automated equipment, imposing strict requirements for model efficiency and lightweight design.

The DFMA model utilizes FasterNet as its backbone network, known for its efficient spatial feature extraction without relying on depthwise separable convolutions. While depthwise separable convolutions offer a lightweight solution, they may fall short in efficiently capturing details within complex structural images. FasterNet’s design, incorporating a combination of Pconv, PWconv, and standard convolution, achieves a balance between lightweight operation and efficiency, making it well-suited for deployment in resource-constrained environments.

Furthermore, DFMA integrates an SPA-ASPP module with EMA (Attention Mechanism), enabling detailed feature capture across multi-scale branches and mitigating the grid effect commonly seen in traditional ASPP modules. The grid effect can lead to feature loss or blurred image boundaries, but the EMA attention mechanism allows the model to focus precisely on key areas of seedlings, such as leaves and stems, resulting in outstanding performance in detail-rich scenarios. This capability is critical for fine-grained segmentation in plant phenotyping, as capturing details aids researchers in better understanding plant growth conditions and morphological characteristics.

### 3.5 Evaluation of the results of the seedling phenotype segmentation experiment

**Figure 13:**
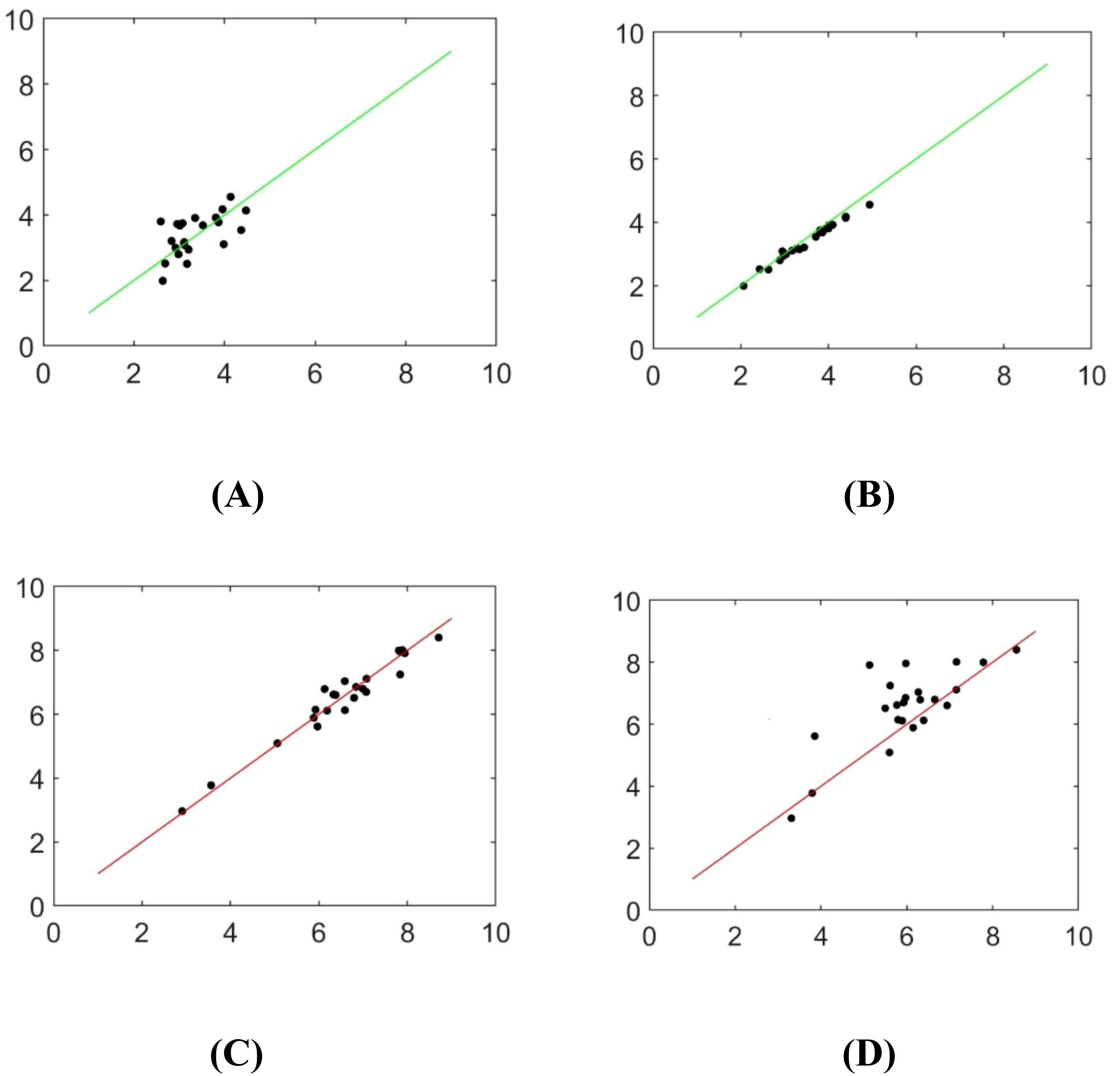
Scatterplot of identifying rice seedlings **(A)** Adam optimizer detects bud length **(B)** SGD optimizer detects bud length **(C)** Adam optimizer detects root length **(D)** The SGD optimizer detects root length.

The skeleton extraction algorithm was employed to identify the central axis of the mask, enabling the computation of the seedling shoot and root length. **Figure 13** and **Table 6** depict the image analysis results obtained through both manual detection and the experimental method described in this paper. In these visualizations, the horizontal axis represents the manually measured values, while the vertical axis represents the corresponding measurements obtained from seedling images using the method outlined in this study. Statistical analysis in **Table 6** is conducted by grouping every 5 seedlings together for assessment.

**Table 6:** Relative errors of different algorithms for length recognition of rice seedling images.

Model	Serial number	Maximum absolute error (cm)	Minimum absolute error (cm)	Mean absolute error (cm)	Improvement (%)	
					Vs. DeepLabV3+	Vs. Unet-VGG
Original Deeplab V3+ model	bud	0.583	0.028	0.386	–	–
	radical	0.506	0.016	0.724		
UNet-VGG	bud	0.876	0.034	0.410	–	–
	radical	1.467	0.074	0.862		
DFMA	bud	0.384	0.007	0.146	+62.20	+64.44
	radical	0.393	0.006	0.231	+68.09	+73.20

The measured values obtained by the algorithm used in this paper and the manual measurement values are highly consistent, and the improved DeepLabv3+ network yields better results than the original DeepLabv3+ network. However, there is still a small error. Possible reasons for the error include the skeleton extraction step after filling the interior of the contour, which causes the algorithm to use the centerline instead of the main root. Additionally, there is an offset in the refinement process, resulting in inconsistent calculated lengths.

Compared to the original DeepLabv3+ model, the improved model reduced the mean absolute errors in measuring shoots and roots by 62.20% and 68.09%, respectively. Compared with the UNet-VGG model, it achieved improvements of 64.44% and 73.20%, respectively, and demonstrated a more significant detection advantage in terms of maximum and minimum absolute errors.

**Figure 14:**
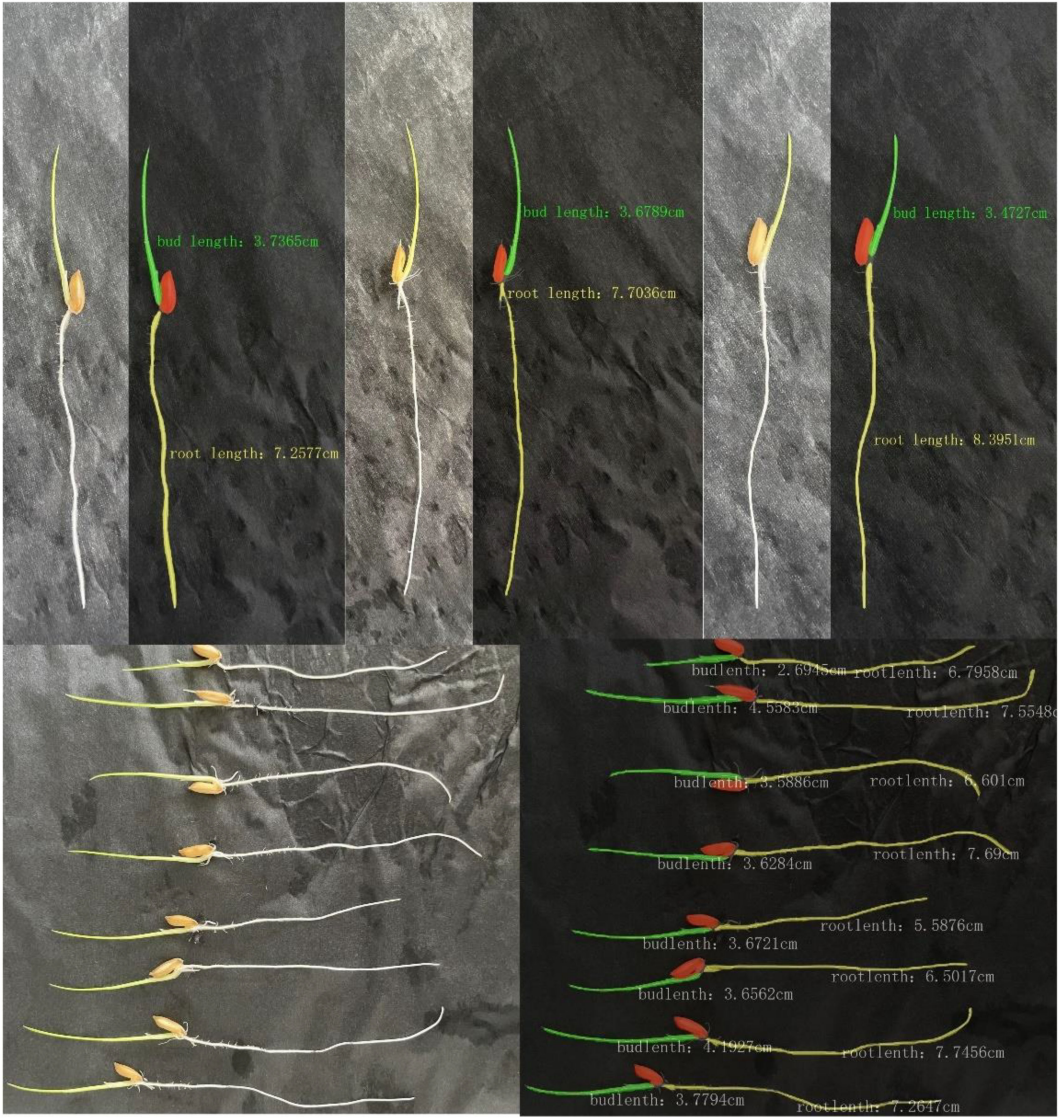
Results of batch testing of rice shoot root lengths.

In this study, based on the improved DeepLabv3+ target segmentation network combined with the length detection algorithm, the sprout target is recognized and segmented, and the sprout length is ultimately obtained. The recognition results are shown in **Figure 14** below. The model in this study demonstrates superior recognition of the target, accurately segments the outline and key parts of the target, and simultaneously avoids confusion between the target and the background. It provides more accurate length detection results and is capable of batch detection.

## 4 Discussion

This study proposes a high-throughput plant phenotyping method based on deep learning, highlighting its broad application potential and significance across multiple fields. Through a non-destructive, efficient, accurate, and consistent measurement approach, we achieved phenotypic analysis of rice seedlings at early growth stages, significantly improving research efficiency and broadening future applications. In line with specific experimental tasks, we selected datasets from four species, three from public Kaggle datasets and one collected independently. This choice allowed us to test the model’s performance under relatively consistent environmental conditions, minimizing external factors and yielding clearer experimental results. However, we recognize that the current datasets are limited in species and environmental diversity, and expanding this diversity is necessary to further enhance the model’s robustness and generalizability. Future research will therefore introduce more samples from diverse species and environmental conditions to improve the model’s adaptability and applicability in complex, dynamic scenarios.

Although the improved DeepLabv3+ and the newly introduced DFMA semantic segmentation model perform excellently in segmentation efficiency and accuracy, they still face limitations in lighting adaptability, cross-crop transferability, and multi-species analysis. To enhance the model’s broad applicability, future work will focus on further strengthening the model’s robustness to varying lighting conditions and exploring ways to adjust feature extraction and attention modules to better accommodate plants with diverse morphological features. As research progresses, we also plan to expand this technology to other crops and plant species, further uncovering growth and developmental characteristics. This will provide scientific support for crop improvement and cultivation, and advance ecological research, helping scientists better understand plant responses to environmental changes.

The application prospects of this technology extend beyond plant phenotyping, with potential in fields such as medical image analysis and autonomous driving, demonstrating deep learning’s immense potential for automation and precision in image processing. This technology holds significant value for research in biology and botany. In the future, we plan to open-source a WeChat-based plant phenotyping mini-program to promote practical applications of this research and facilitate further developments. This will provide innovative tools and directions for plant breeding and crop improvement.

## 5 Conclusion

In summary, our study addresses a critical need in the rapidly evolving field of plant phenotypic research. Accurate seedling length measurement is essential for evaluating seed viability and growth status. We have developed an efficient and versatile deep learning approach, named DFMA, which incorporates the innovative PSPA-ASPP structure. Our model consistently outperforms traditional methods and other models, achieving remarkable segmentation and detection results across various plant species. DFMA generates precise segmentation masks that highlight detailed developmental aspects of seedling components, such as cotyledons, hypocotyls, and roots. Furthermore, we introduce a novel seedling length measurement algorithm, providing precise parameters for a comprehensive plant phenotypic analysis. Our research holds great promise for offering more efficient tools and data support to advance the field of plant biology, enhancing our understanding of plant genetics and growth trends in the top-tier scientific community.

## Data Availability

The raw data supporting the conclusions of this article will be made available by the authors, without undue reservation.
